# Different Targets of Monoclonal Antibodies in Neuromyelitis Optica Spectrum Disorders: A Meta-Analysis Evidenced From Randomized Controlled Trials

**DOI:** 10.3389/fneur.2020.604445

**Published:** 2020-12-17

**Authors:** Tao Xue, Jiahao Yu, Shujun Chen, Zilan Wang, Yanbo Yang, Zhouqing Chen, Zhong Wang

**Affiliations:** ^1^Department of Neurosurgery & Brain and Nerve Research Laboratory, The First Affiliated Hospital of Soochow University, Suzhou, China; ^2^Department of Thoracic Surgery, The First Affiliated Hospital of Soochow University, Suzhou, China; ^3^Department of Neurology, The First Affiliated Hospital of Soochow University, Suzhou, China

**Keywords:** neuromyelitis optica spectrum disorders, monoclonal antibody, meta-analysis, aquaporin-4 autoantibody, rct

## Abstract

**Background:** Neuromyelitis optica spectrum disorder (NMOSD), an autoimmune inflammatory disorder of the central nervous system, often leads to vision loss or paralysis. This meta-analysis focused on the assessment of the monoclonal antibody therapy in NMOSD and compared different targets of monoclonal antibodies with each other in terms of efficacy and safety outcomes.

**Method:** We searched through the databases of MEDLINE, EMBASE, Central Register of Controlled Trials (CENTRAL), and clinicaltrials.gov for randomized controlled trials (RCTs) evaluating monoclonal antibody therapy in NMOSD up to April 2020.

**Results:** We identified seven randomized controlled trials (RCTs), including 775 patients (monoclonal antibody group, *n* = 485 and placebo group, *n* = 290). Monoclonal antibody therapy decreased relapse risk (RR 0.33, 95% CI 0.21–0.52, *P* < 0.00001), annualized relapse rate (ARR) (mean −0.28, 95% CI −0.35−0.20, *P* < 0.00001), expanded disability status scale score (EDSS) (mean −0.19, 95% CI −0.32−0.07, *P* = 0.002) and serious adverse events (RR 0.78, 95% CI 0.61–1.00, *P* = 0.05). However, we did not observe any significant difference in terms of adverse events or mortality. Further, the subgroup analysis demonstrated that the anti-complement protein C5 monoclonal antibody (eculizumab) might have a lower relapse risk (RR 0.07, 95% CI 0.02–0.23, *P* < 0.0001) in the AQP4 seropositive patients, and anti-interleukin-6 receptor monoclonal antibodies (satralizumab and tocilizumab) showed decreased EDSS score (mean −0.17, 95% CI −0.31−0.02, *P* = 0.02) more effectively than other monoclonal antibodies.

**Conclusions:** Monoclonal antibodies were effective and safe in NMOSD. Different targets of monoclonal antibodies might have their own advantages.

## Key Points

- Monoclonal antibody therapy was effective and safe in NMOSD treatment.
- Eculizumab might have a lower relapse risk in the AQP4 seropositive patients.
- Satralizumab and tocilizumab might decrease the EDSS score more effectively.

## Introduction

Neuromyelitis optica spectrum disorder (NMOSD) is a relapsing inflammatory autoimmune disease of the central nervous system whose symptoms are associated with optic nerve, spinal cord, brain stem, and cerebrum injury. The clinical manifestations of patients are usually: (a) optic nerve attacks including loss of vision or blindness; (b) spinal cord attacks including severe motor impairment or even the loss of the ability to walk, sensory impairment, and bowel/bladder dysfunction; (c) brain stem attacks including refractory nausea, vomiting, and burping; (d) cerebrum attacks including cognitive impairment, language dysfunction, and drowsiness (1–5). Aquaporin-4 (AQP4) antibody seropositive patients accounted for 80% among all NMOSD patients (6). Recently, antibody to myelin oligodendrocyte glycoprotein (MOG) was considered as another NMOSD marker in AQP4 negative patients (7). However, more experimental data is needed to comprehensively illustrate such results (2).

At present, the primary goals for treating NMOSD are restricted to reduce severity of acute attack and prevent relapse in remission (8). The treatment for acute episodes mainly includes corticosteroids, intravenous immunoglobulin and plasma exchange therapy. In addition, to reduce relapse risk immunosuppressive drugs such as azathioprine (AZA), mycophenolate mofetil (MMF), and monoclonal antibodies like rituximab are frequently used in clinical practices (8–11). However, few studies have reported unavoidable adverse reactions on the patients with NMOSD, and these were treated with long-term immunosuppressive drugs (12–14). Therefore, new monoclonal antibodies have become popular and many studies now shifted their attention on them.

Monoclonal antibodies which were widely used for NMOSD in clinical trials mainly include: rituximab, eculizumab, inebilizumab, satralizumab, tocilizumab, etc (15–21). Rituximab is a chimeric monoclonal antibody against human CD20. It is an effective drug for NMOSD patients, especially in AQP4 seropositive patients (22, 23). Inebilizumab (MEDI-551) is a humanized monoclonal antibody against the CD19 B cell protein extracellular ring of the IgG1 subtype. Previous studies have reported that inebilizumab has potential application value for patients with NMOSD due to the existence of a similar mechanism to that of rituximab (24). Satralizumab and tocilizumab are both humanized recombinant monoclonal antibodies targeting interleukin-6 receptor (IL-6R), however, according to previous studies satralizumab has better pharmacokinetics than tocilizumab via antibody recovery technique. Further, based on previous clinical trials, satralizumab, and tocilizumab both reduced the NMOSD relapse risk (25–27), while satralizumab appeared to have no effects on reducing the pain and fatigue of patients (27). Eculizumab can reduce the damage related with the inflammatory response to the nervous system by inhibiting the complement protein C5 and blocking terminal complement activation (28). One of the studies carried out by Pittock et al. (29) declared that eculizumab reduced the relapse risk of AQP4 seropositive patients compared with placebo groups.

The effectiveness and safety of monoclonal antibodies have not been systematically evaluated in prospective series or randomized clinical trials. Therefore, still several issues are remaining to be resolved, including whether monoclonal antibodies can decrease relapse risk, annualized relapse rate (ARR), and the Expanded Disability Status Scale (EDSS) score of NMOSD patients with no further enhancement in adverse events, serious adverse events and mortality. Therefore, we conducted a meta-analysis of pooled data from the seven RCTs to investigate the significance of monoclonal antibodies for NMOSD and to explore the potential factors that might influence the efficacy and safety of monoclonal antibodies.

## Method

### Study Protocol

We drafted a study protocol by following the Cochrane Collaboration format at the beginning of the projects (30).

### Eligibility Criteria

Only studies that meet the following criteria can be adopted in this paper: (a) Type of study: RCT; (b) Language restrictions: English only; (c) Participating patients: NMOSD patients; (d) Intervention: monoclonal antibody; (e) Efficacy Outcomes: Relapse risk on trial, ARR, and EDSS score change; (f) Safety Outcomes: adverse events, serious adverse events as well as mortality. Exclusion criteria are as follows: (a) Research Type: case reports, cohort studies, case reviews and retrospective studies; (b) Control: active control (i.e., that a known, effective treatment as opposed to a placebo is compared to an experimental treatment).

### Information Sources and Search Strategy

There were two independent authors (TX and JY) searching data systematically form the four databases: MEDLINE, EMBASE, Central Register of Controlled Trials (CENTRAL), and https://clinicaltrials.gov./ The following search strategy was used: (((Monoclonal antibody[Title/Abstract])) AND (Neuromyelitis Optica Spectrum Disorders[Title/Abstract])) OR (Devic's disease[Title/Abstract]) for MEDLINE; “Monoclonal antibody”/exp AND “Neuromyelitis Optica Spectrum Disorders”/exp OR “Devic's disease”/exp for EMBASE; “Monoclonal antibody” in Title Abstract Keyword AND “Neuromyelitis Optica Spectrum Disorders” in Title Abstract Keyword OR “Devic's disease” in Title Abstract Keyword for CENTRAL; “Monoclonal antibody | Neuromyelitis Optica Spectrum Disorders or Devic's disease” for clinicaltrials.gov. Studies that matched the abstracts and titles were queried. Only clinical trials, meta-analysis, reviews and case reports were included in the search. In addition, two authors (TX and JY) independently searched the paper and data to make sure all relevant studies were included in the search in April 2020.

### Study Selection and Data Collection

Relevant studies screened from MEDLINE, EMBASE, CENTRAL, and clinicaltrials.gov were evaluated by two authors (TX and JY) independently in April 2020. When disagreements emerged among two reviewers, the disputed data was discussed with the third person (SC), who did not participate in the data collection, to determine whether these data should be included in the study. The important baseline data (Table 1) including: names and mechanisms of monoclonal antibodies; publications, phases and regions of studies; gender composition, AQP4 serology, nd add-on drugs of patients were extracted from RCTs by rigorous selection and evaluation.

**Table 1:** Characteristics of the included studies.

**Study**	**Monoclonal antibody**	**Mechanism**	**Publications**	**Phase**	**Regions**	**Treatment group, (No. of participants)**		**Male (%)**		**Mean age** **±** **SD (year)**		**AQP4 seropositive (%)**		**Add-on drugs**
						**mAb**	**Placebo**	**mAb**	**Placebo**	**mAb**	**Placebo**	**mAb**	**Placebo**	
Nikoo et al. (23) (NCT03002038)	Rituximab	CD20 B cell depletion	Journal of neurology	III	1 center in Iran	33	35(AZA)	12.1	20	35.33 ± 8.98	32.35 ± 9.56	39.4	57.1	Azathioprine (AZA) and prednisolone in placebo group.
Pittock et al. (29) (NCT01892345)	Eculizumab	C5 complement inhibitor	New England journal of medicine	III	70 centers in 18 countries	96	47	8	11	35.8 ± 14.03	38.5 ± 14.98	100	100	Immunosuppressive drugs in both group.
Cree et al. (24) (NCT02200770)	Inebilizumab	CD19 B cell depletion	Lancet	II / III	99 centers in 25 countries	174	56	9	11	43.0 ± 11.6	42.6 ± 13.9	93	93	Prednisone in both group.
Yamamura et al. (27) (NCT02028884)	Satralizumab	Interleukin-6 receptor blocker	New England journal of medicine	III	34 centers in 11 countries	41	42	10	5	40.8 ± 16.1	43.4 ± 12.0	66	67	AZA, mycophenolate mofetil, glucocorticoids in both group.
Tahara et al. (22) (UMIN000013453)	Rituximab	CD20 B cell depletion	Lancet neurology	II	8 centers in Japan	19	19	11	0	53	47	74	68	Oral glucocorticoids in both group.
Traboulsee et al. (25) (NCT02073279)	Satralizumab	Interleukin-6 receptor blocker	Lancet neurology	III	44 centers in 13 countries	63	32	27	3	36.4 ± 10.7	39.3 ± 13.3	65	72	None.
Zhang et al. (26) (NCT03350633)	Tocilizumab	Interleukin-6 receptor blocker	Lancet neurology	II	6 centers in China	59	59(AZA)	7	10	48.1 ± 13.4	45.3 ± 14.5	85	90	Azathioprine (AZA) in placebo group.

### Risk of Bias

We used Review Manager 5.3 software to assess the risk of bias for each study. There were some biases including attrition bias, reporting bias, detection bias, selection bias, performance bias, and other potential biases. We applied the unified standard of the Cochrane Collaboration to evaluate the risk of bias of RCTs.

### Summary Measures and Synthesis of Results

The data was assessed by Review Manager 5.3 software. The dichotomous outcomes were calculated and analyzed by a random effect model which appeared as a risk ratio [relative risk (RR); 95% confidence interval (CI)]. We use *I*^2^ statistic to estimate heterogeneity. The *I*^2^ statistic as follows: *I*^2^ <30% means “low heterogeneity,” 30% < *I*^2^ <50% represents “moderate heterogeneity,” *I*^2^ >50% denotes “substantial heterogeneity” (31). Due to the different pharmacological effects of the monoclonal antibody therapy, we divided the monoclonal antibody into three subgroups. They were anti-B cells monoclonal antibodies, anti-interleukin-6 (IL-6) receptor monoclonal antibodies, and complement protein C5 inhibitor monoclonal antibodies. A sensitivity analysis was used to discuss the stability of the consolidated results. Two-tailed tests were performed in all analyses. A *P* < 0.05 was considered to be significant for all analyses.

## Results

### Study Identification and Selection

**Figure 1:**
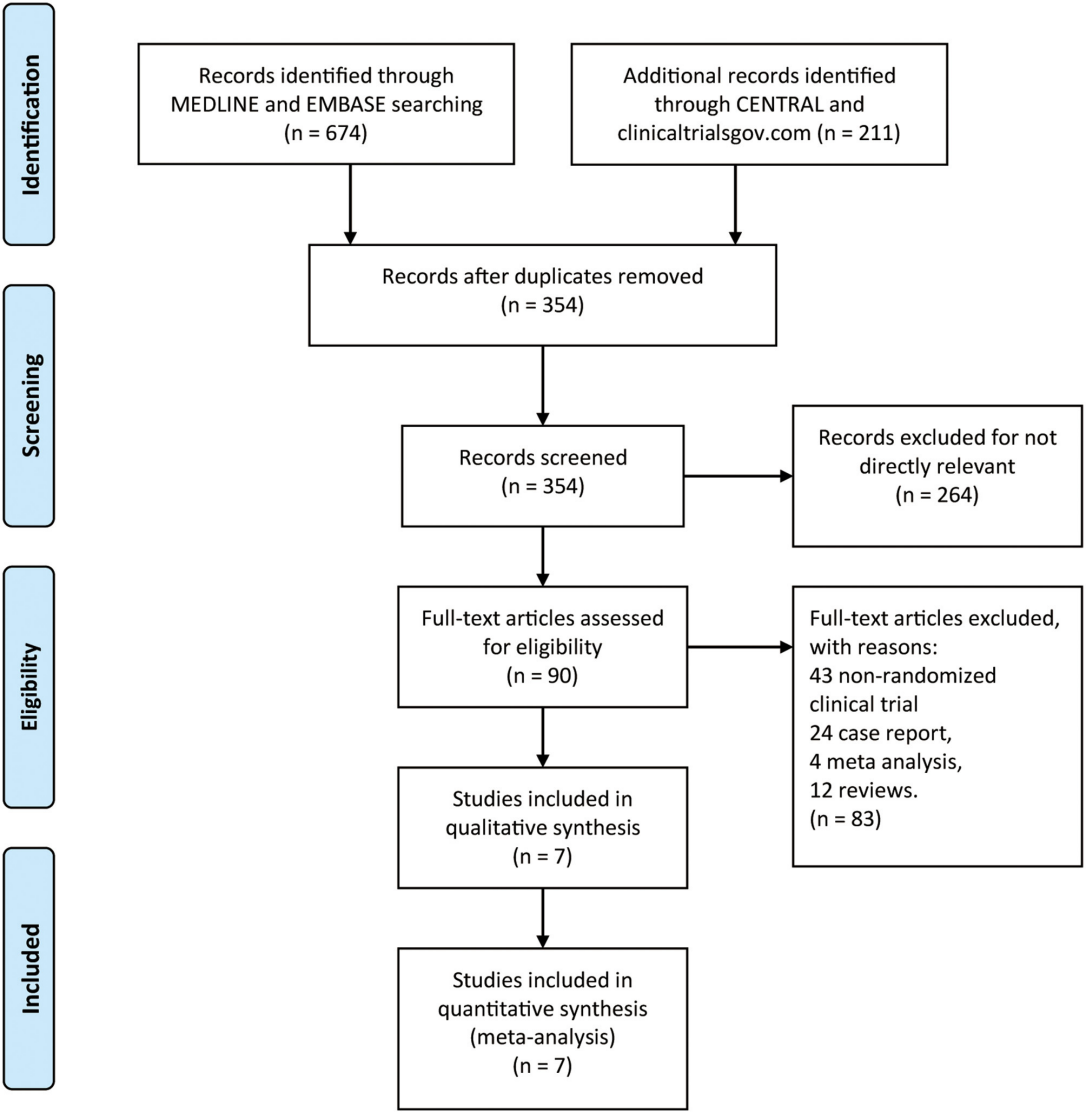
The study search, selection, and inclusion process.

By searching MEDLINE, EMBASE, CENTRAL, and clinicaltrials.gov database, we identified 885 records. After removing duplicates, there were 354 records left (Figure 1). In addition, remaining 264 records were not directly relevant. Seven RCTs (22–27, 29) finally contained 775 patients (485 in monoclonal antibody group: mean age 41.02, 11.34% male, and 88.66% female, 83.01% AQP4 seropositive and 16.99% AQP4 seronegative; 290 in placebo group: mean age 41.29, 9.31% male, and 90.69% female, 81.38% AQP4 seropositive and 18.62% AQP4 seronegative) which were included in qualitative synthesis. The main baseline information of the seven RCTs is illustrated in Table 1.

### Efficacy Outcome

**Figure 2:**
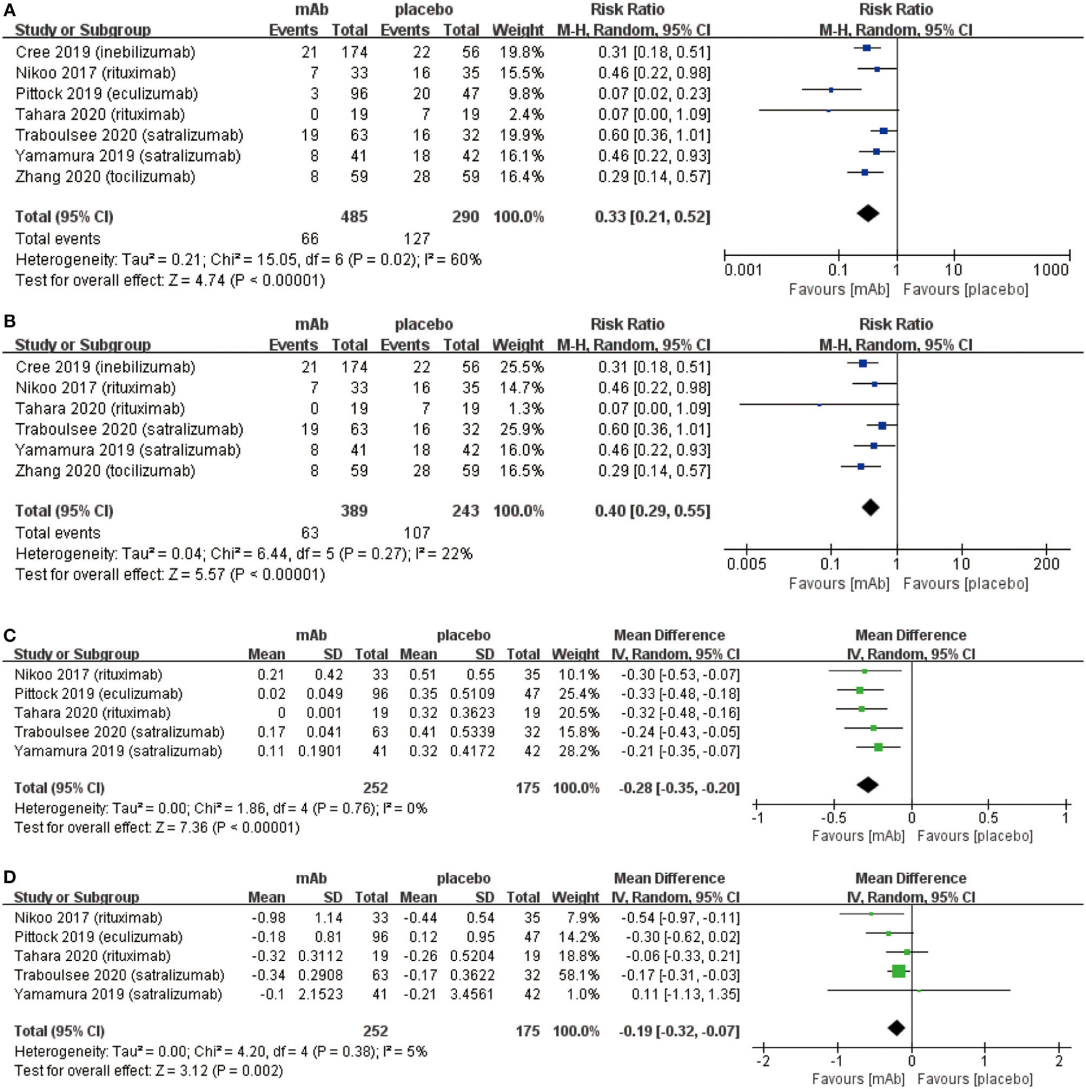
The pooled relative risk of the efficacy outcomes. The blue diamond indicates the estimated relative risk (95% confidence interval) and the green diamond indicates the mean difference (95% confidence interval) for all patients together. **(A)** on-trial relapse risk. **(B)**, on-trial relapse risk without Pittock et al. (29). **(C)**, ARR. **(D)**, EDSS score change.

We attributed the efficacy outcomes of the treatment results to three factors as follows: (a) relapse risk; (b) ARR; (c) EDSS score change. At first, the on-trial relapse risk (RR 0.33, 95% CI 0.21 to 0.52, *P* < 0.00001; Figure 2A) was lower in the monoclonal antibody group than that in the placebo group. However, the heterogeneity of relapse risk was as high as 60%. To find the source of heterogeneity, we carried out a sensitivity analysis (Supplementary Figure 1) which showed stable consolidated data. In addition, we observed that when data from Pittock et al. (29) was excluded, the heterogeneity of relapse risk (Figure 2B) dropped to 22%. This indicated that the particularly low relapse risk of Eculizumab group in the study of Pittock et al. (29) led to the high heterogeneity. Further, the monoclonal antibody group recovered with a lower ARR (mean −0.28, 95% CI −0.35−0.20, *P* < 0.00001; Figure 2C) than the placebo group. Finally, the change related to the EDSS score (mean −0.19, 95% CI −0.32−0.07, *P* = 0.002; Figure 2D) of patients in the monoclonal antibody group decreased significantly compared with the placebo group.

### Safety Outcome

**Figure 3:**
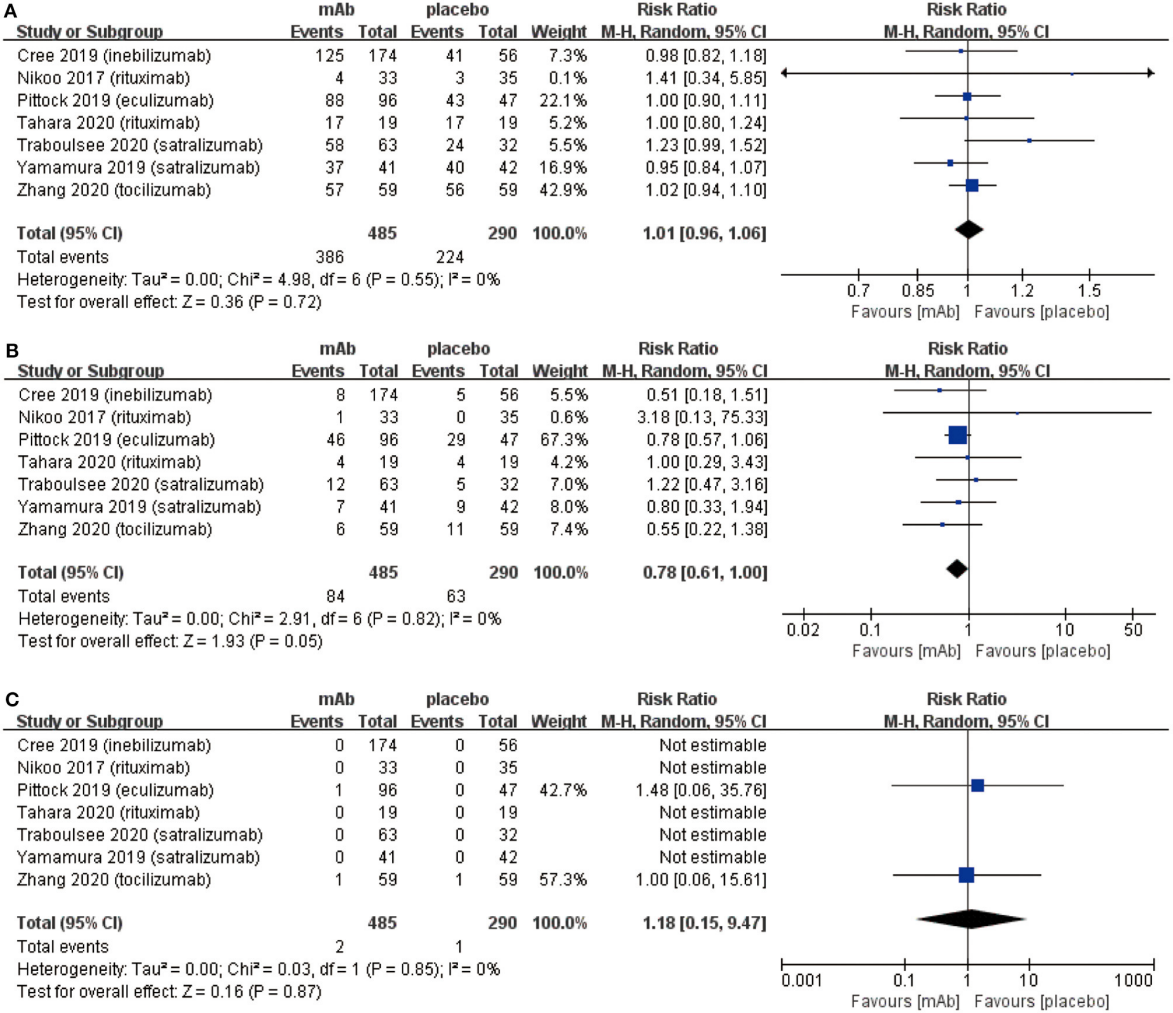
The pooled relative risk of the safety outcomes. The diamond indicates the estimated relative risk (95% confidence interval) for all patients together. **(A)** adverse events. **(B)** Serious adverse events. **(C)** Death rate.

From the aspect of safety outcomes, we mainly considered the following three factors: (a) adverse events, (b) serious adverse events, and (c) mortality. Initially, there were no significant differences observed in adverse events (RR 1.01, 95% CI 0.96–1.06, *P* = 0.72; Figure 3A) between the monoclonal antibody group and placebo group. Adverse events mainly included: infusion related reactions, pain (limb, joint, or back), nasopharyngitis, and infection (upper respiratory or urinary tract), etc. However, the frequency of serious adverse events (RR 0.78, 95% CI 0.61–1.00, *P* = 0.05; Figure 3B) might have a downward trend in the monoclonal antibody group. Serious adverse events were included in the adverse events. These were different from adverse events in that serious adverse events could interrupt the patient's daily activities and may lead to systemic medication or other treatment. Serious adverse events were able to incapacitate patients. Eventually, NMOSD patients had a very low mortality (3/775) in 7 included RCTs and no statistically significant difference was observed in mortality from the monoclonal antibody group to the placebo group (RR 1.18, 95% CI 0.15–9.47, *P* = 0.87; Figure 3C).

### Subgroup Analysis

**Figure 4:**
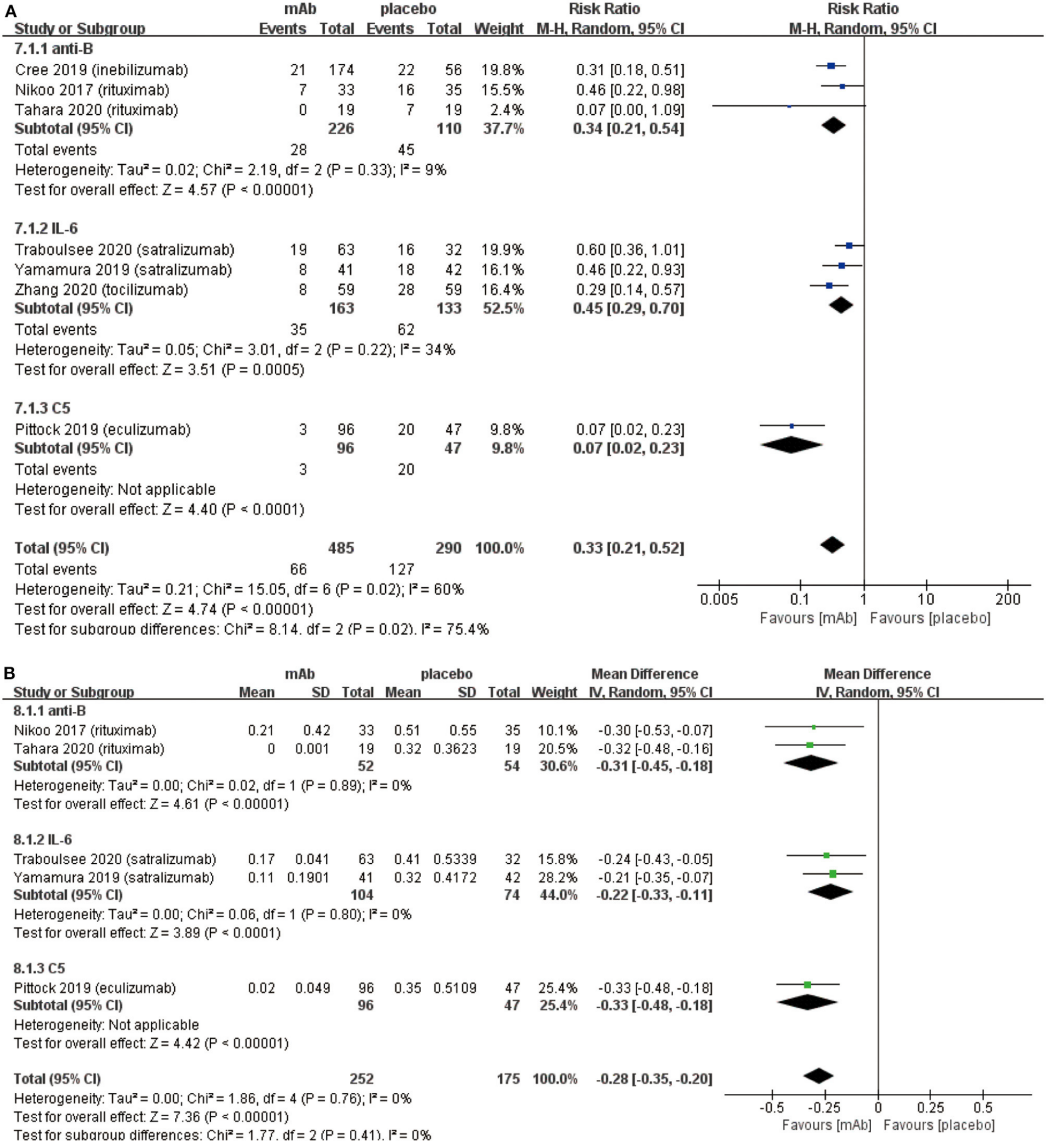
Subgroup analysis of effect of monoclonal antibodies with different targets, the blue diamond indicates the estimated relative risk (95% confidence interval) and the green diamond indicates the mean difference (95% confidence interval) for all patients together. **(A)** on-trial relapse risk in subgroup. **(B)** ARR in subgroup. anti-B, anti-B cell monoclonal antibodies; IL-6, anti-interleukin-6 receptor monoclonal antibodies; C5, anti-complement protein C5 monoclonal antibody.

**Figure 5:**
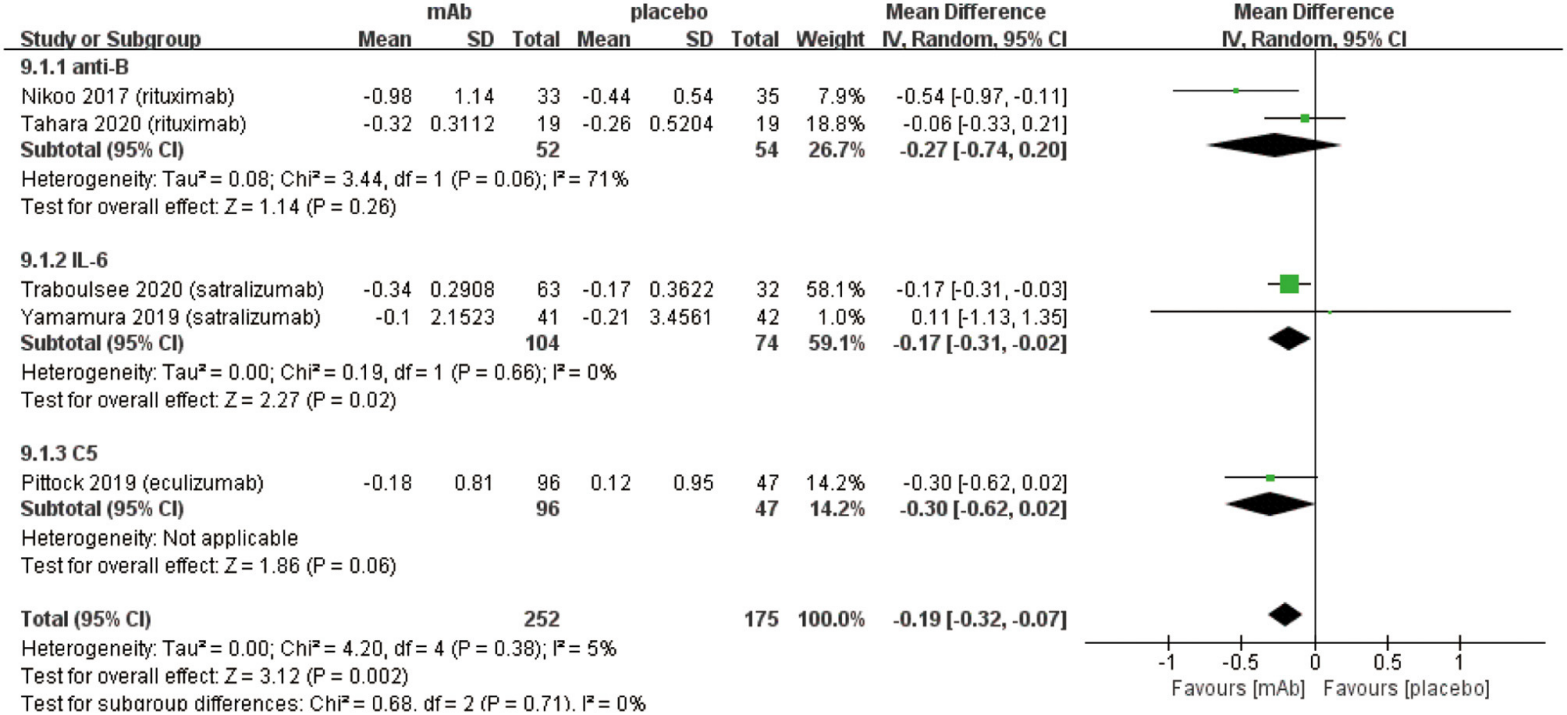
Subgroup analysis of EDSS score change of monoclonal antibodies with different targets, the diamond indicates the mean difference (95% confidence interval) for all patients together. anti-B: anti-B cell monoclonal antibodies, IL-6: anti-interleukin-6 receptor monoclonal antibodies, C5: anti-complement protein C5 monoclonal antibody.

**Figure 6:**
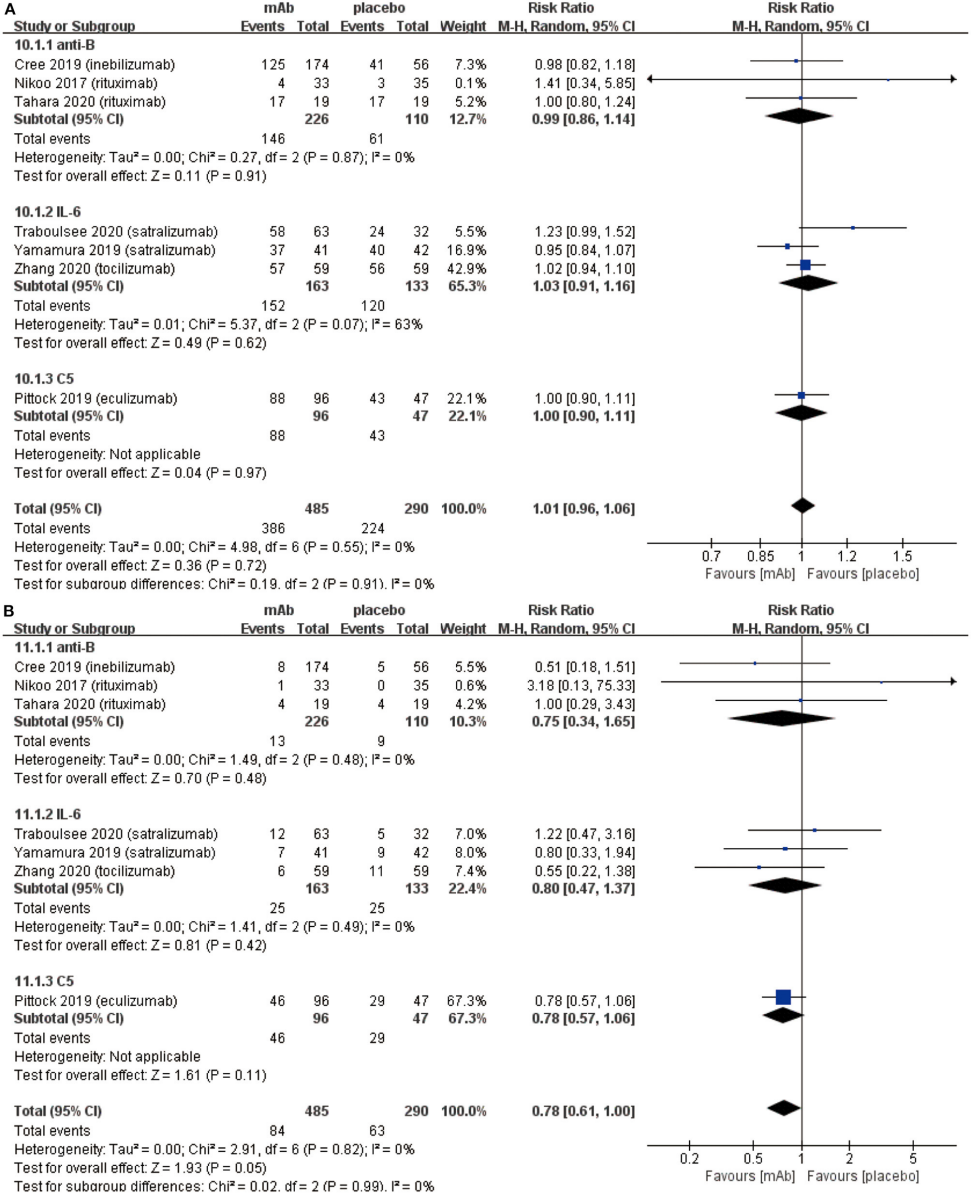
Subgroup analysis of safety of monoclonal antibodies with different targets, the diamond indicates the estimated relative risk (95% confidence interval) for all patients together. **(A)** adverse events in subgroup. **(B)** serious adverse events in subgroup. anti-B, anti-B cell monoclonal antibodies; IL-6, anti-interleukin-6 receptor monoclonal antibodies; C5, anti-complement protein C5 monoclonal antibody.

We established a subgroup to evaluate the efficacy and safety in different pharmacological effects of monoclonal antibodies. Further, monoclonal antibodies were divided into three subgroups depending on the different targets: (a) anti-B cell monoclonal antibodies (anti-B) including rituximab and inebilizumab; (b) anti-interleukin-6 receptor monoclonal antibodies (IL-6) including: satralizumab and tocilizumab; (c) anti-complement protein C5 monoclonal antibody (C5) including eculizumab. Initially, eculizumab showed lower relapse risk (anti-B: RR 0.34, 95% CI 0.21–0.54, *P* < 0.00001; IL-6: RR 0.45 95% CI 0.29–0.70, *P* = 0.0005; C5: RR 0.07, 95% CI 0.02–0.23, *P* < 0.0001; Figure 4A) than other monoclonal antibodies. It is worth mentioning that patients treated with eculizumab in the study of Pittock et al. were all AQP4 seropositive. Probably, it was a better choice for AQP4 seropositive patients to be treated by eculizumab. More trials are needed to confirm this result from Pittock et al. In addition, there were no significant differences observed in ARR (anti-B: RR −0.31, 95% CI −0.45−0.18, *P* < 0.00001; IL-6: RR −0.22, 95% CI −0.33−0.11, *P* < 0.0001; C5: RR −0.33, 95% CI −0.48−0.18, *P* < 0.00001; Figure 4B) among subgroups. From the perspective of the EDSS score change (anti-B: RR −0.27, 95% CI −0.74–0.20, *P* = 0.26; IL-6: RR −0.17, 95% CI −0.31−0.02, *P* = 0.02; C5: RR −0.30, 95% CI −0.62–0.02, *P* = 0.06; Figure 5), we detected that anti-interleukin-6 receptor monoclonal antibodies exhibited significantly a better performance to improve functional recovery than other monoclonal antibodies. When it comes to adverse events (anti-B: RR 0.99, 95% CI 0.86–1.14, *P* = 0.91; IL-6: RR 1.03, 95% CI 0.91–1.16, *P* = 0.62; C5: RR 1.00, 95% CI 0.90–1.11, *P* = 0.97; Figure 6A) and serious adverse events (anti-B: RR 0.75, 95% CI 0.34–1.65, *P* = 0.48; IL-6: RR 0.80, 95% CI 0.47–1.37, *P* = 0.42; C5: RR 0.78, 95% CI 0.57–1.06, *P* = 0.11; Figure 6B), no apparent differences were observed among different subgroups.

### Risk of Bias in Included Studies

**Figure 7:**
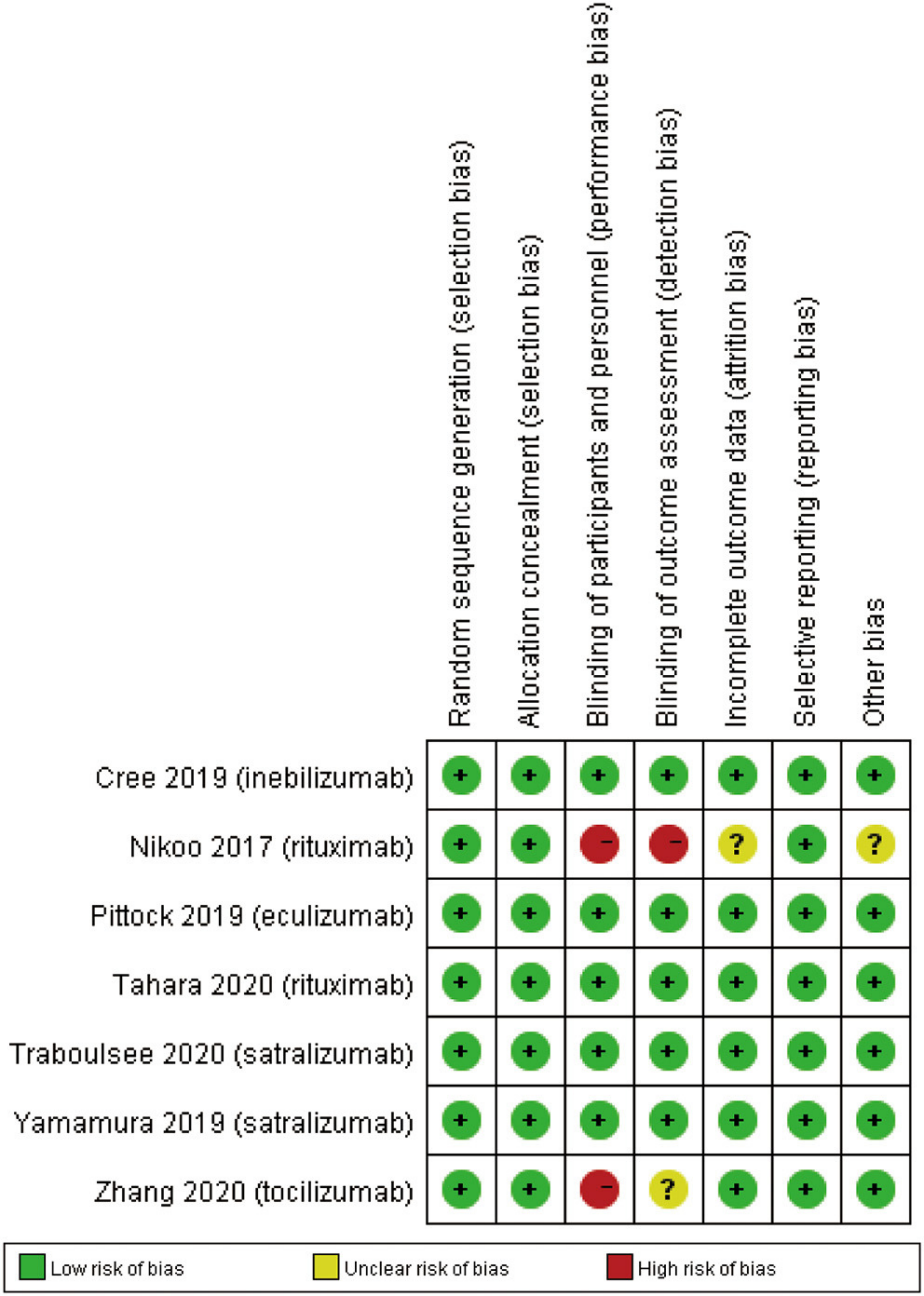
Risk of bias: A summary table for each risk of bias item for each study.

The details of risk bias for 7 RCTs were showed in Figure 7. All RCTs showed low risk of biases in the random sequence generation, allocation concealment and selective reporting. For the blinding of participants and personnel, the risk of bias was high in 2 RCTs. For the blinding of outcome assessment, the risk of bias was high in Nikoo et al. (23) and unclear in Zhang et al. (26). In addition, Nikoo et al. (23) had unclear risk of incomplete outcome data and other bias.

## Discussion

Based on the results of this meta-analysis, we consider that monoclonal antibody therapy is effective and safe for the treatment of NMOSD.

Disability of NMOSD patients which can primarily be assessed using the EDSS score, are usually caused by irreversible damage to the nervous system after recurrent attacks (32). The primary goals of NMOSD treatment are to control the symptoms including inflammatory response during acute episodes and reduce the relapse risk in remissive stage (8). Therefore, generally for the relapse risk, ARR and EDSS score changes are used as indicators of the efficacy outcomes. Our meta-analysis illustrated that monoclonal antibody therapy has significant benefits in preventing recurrence. As shown in Figure 2A, we concluded that monoclonal antibodies reduce the on-trial relapse risk but heterogeneity was as high as 60%. The substantial heterogeneity was likely from the different baseline characteristics such as different monoclonal antibodies, pharmacological mechanisms, AQP4 serology and add-on drugs. After excluding each RCT respectively, we found the superior effect of eculizumab in the study of Pittock et al. (29) that produced such high heterogeneity. When this RCT was excluded, the heterogeneity of relapse risk dropped to 22% (Figure 2B). Additionally, it is worth mentioning that only patients in the Pittock et al. (29) were all AQP4 seropositive. Moreover, subgroup analysis based on different pharmacological mechanisms were carried out and the heterogeneity in each subgroup was observed to be in the acceptable form (Figure 4A). Furthermore, we performed a sensitivity analysis on relapse risk (Supplementary Figure 1) which showed stable consolidated data. Therefore, we consider that the results obtained for of relapse risk were reliable. In addition, we discovered that monoclonal antibodies can reduce ARR and disability. One of the main reasons for improving neurological function might be related to monoclonal antibodies lowering the relapse risk of NMOSD patients so as to attenuate the damage of recurrent attacks. In spite of the statistical absence for ARR and EDSS score change in 2 RCTs (24, 26), we can still rely on monoclonal antibody therapy which is indeed able to reduce ARR and EDSS score in NMOSD patients. Nonetheless, from these obtained results, we suggest that monoclonal antibody therapy should be recommended to NMOSD patients, whether patient is AQP4 seropositive or not to prevent relapse and improve functional recovery.

In the present study, as for the safety outcomes, more attention was paid to adverse events and serious adverse events because NMOSD showed an extremely low mortality rate in 7 RCTs. Further, no significant difference was observed (Figure 3A) between monoclonal antibody group and placebo group for the adverse events. The main adverse events were: infection (upper respiratory tract or urinary tract), headache, infusion-related reaction, nasopharyngitis pain (limb, joint, or back), etc. Interestingly, our meta-analysis exhibited monoclonal antibody therapy might have a tendency to reduce serious adverse events (Figure 3B). Types of serious adverse events were very various, however, we found part of serious adverse events caused by relapse and hence the results could be explained by the relapse-preventing function of monoclonal antibodies. Additionally, monoclonal antibodies may cause some specific adverse events due to their special pharmacological mechanisms. Initially, anti-B cell monoclonal antibodies such as rituximab and inebilizumab can cause damage to B cells and reduce the human's immune function. It was also reported to increase the risk of cancer and infections (33). However, only two patients were diagnosed with malignant tumors in the seven RCTs [uterine cancer in Tahara et al. (22) and multiple myeloma in Zhang et al. (26)]. The studies carried out by Tahara et al. and Zhang et al. claimed that the occurrence of malignant tumors was not related to their treatment by monoclonal antibodies. Further, previous studies also reported the existence of a risk factor for invasive meningococcal disease among patients who received eculizumab despite receipt of meningococcal vaccine (12). However, we found only one case of meningococcal septicemia after vaccination during the treatment of eculizumab in the study of Pittock et al. (29). Subsequently, the anti-IL6R monoclonal antibodies such as satralizumab and tocilizumab resulted in dyslipidemia, however, it did not increase risk of cardiovascular or cerebrovascular diseases (34). Based on these safety outcomes, we concluded that monoclonal antibody therapy is safe and even safer than non-monoclonal antibody therapy.

Subgroup analysis were created to compare the monoclonal antibodies of three different targets. Initially, anti-B cell monoclonal antibodies (anti-B) included rituximab, and inebilizumab (MEDI-551). Rituximab can bind to CD20 epitopes expressed by prep and mature B cells to cause the destruction of B cells (35). It was reported that rituximab has acceptable tolerance, reduces the relapse frequency, and improves disability in most patients with NMOSD (36, 37). The target of inebilizumab is the CD19 epitope also expressed by B cells. Compared with rituximab, inebilizumab can damage B cells more broadly, and remove the plasma blasts that produce AQP4-antibodies or MOG-antibodies (38). The second subgroup named anti-interleukin-6 receptor monoclonal antibodies (IL-6) consisted of satralizumab and tocilizumab. These antibodies are humanized reconstituted monoclonal antibodies which target the IL-6 receptor (IL-6R) and have the same pharmacological mechanism. When Satralizumab and tocilizumab are combined with IL6-R, they prevent the differentiation of inflammatory Th17 cells and plasma blasts. The difference between the two monoclonal antibodies is that satralizumab was designed to improve pharmacokinetics by applying the “antibody recycling technology” to tocilizumab (39). The third subgroup consisted of anti-complement protein C5 monoclonal antibody (C5). This group only had eculizumab which can bind to C5 to inhibit its cleavage into C5a and C5b to prohibit complement activation (40).

We conducted the subgroup analysis to detect whether there were any differences in efficacy and safety outcomes among above-mentioned three kinds of monoclonal antibodies. The results revealed that eculizumab might be better at preventing relapse (Figure 4A) than other monoclonal antibodies. Earlier, NMOSD was considered as an inflammatory autoimmune disease related to the central nervous system. However, through pathological results, it was reported that NMOSD (at least AQP4 seropositive patients) was an astrocytic lesion leading to oligodendrocyte injury and demyelination (41). The cause of astrocyte injury might be the antibody-dependent cytotoxicity induced by the complements activation which resulted from binding AQP4-IgG to their targets (42). In addition, complementary anaphylatoxins (including C5a and C4a) played an important role in aggravating the inflammatory response (43). Patients treated with eculizumab in the study of Pittock et al. (29) were all AQP4 seropositive. Based on the latest pathogenesis reports, eculizumab, as anti-complement protein C5 monoclonal antibodies, is probably able to avoid complementary activation, reduce astrocyte injury, demyelination, and prevent recurrence in AQP4 seropositive patients. Therefore, until now eculizumab is probably the best choice for AQP4 seropositive patients to prevent relapse. However, more trials are required for eculizumab in AQP4 seropositive to confirm this hypothesis. Besides, another speculation should be also put forward: eculizumab might have a better therapeutic effect not only on AQP4 seropositive patients but also on AQP4 seronegative patients. The therapeutic effect for patients with seronegative is still unclear. Therefore, clinical trials for eculizumab including patients with mixed AQP4 serology are needed to confirm these assumptions.

Compared with other monoclonal antibodies, anti-interleukin-6 receptor monoclonal antibodies (IL-6) reduced EDSS score (Figure 5) more effectively in NMOSD patients. It was reported that IL-6 increases blood-brain barrier permeability; anti-interleukin-6 receptor monoclonal antibody specifically binds to soluble membrane interleukin-6 receptors and inhibits IL-6 signal transduction (38). The weaker inflammatory response results in less damage to the central nervous system after using anti-interleukin-6 receptor monoclonal antibody. Hence, the degree of disability assessed by the EDSS score was lower than other monoclonal antibodies. In addition, no statistically significant differences were observed among 3 kinds of monoclonal antibodies in terms of ARR, adverse events and serious adverse events (Figures 4B, 6A,B). Nonetheless, more clinical trials which can evaluate three types of monoclonal antibodies with each other on their efficacy and safety outcomes need to be conducted.

In terms of the current situation of monoclonal antibody therapy for NMOSD, only rituximab was widely used in clinical practice. However, initially, merely some open-label, non-controlled, and non-randomized observational studies provided evidence of the efficacy of rituximab in the treatment of NMOSD. Some data showed that the percentage of patients treated with rituximab for 12–60 months without recurrence ranges from 33 to 100% (16, 35, 44–52). It was the powerful efficacy of rituximab that led to rituximab being increasingly used in first-line treatment (16, 19). Recently, eculizumab has been approved by the US Food and Drug Administration (FDA) for the treatment of NMOSD and it will gradually enter the field of clinical treatment. Some of the other monoclonal antibodies mentioned in this article, such as inebilizumab, satralizumab, and tocilizumab etc. have not been used in clinical treatment and are still in clinical trials at the time of writing. Meanwhile, other monoclonal antibodies which have not been assessed by randomized controlled trials might have great potential to treat NMOSD patients. Natalizumab, a monoclonal anti-body against the adhesion molecule α4 integrin (CD49d), can interfere with the entry of B cells into the central nervous system (53), which is believed to be the theoretical basis for the treatment of NMOSD. However, the study carried out by Lee et al. (54) and Kleiter et al. (55) deemed that natalizumab might not work effectively on patients with NMOSD (54, 55). Aquaporumab, a non-pathogenic recombinant human monoclonal antibody against AQP4 protein, can prevent the pathogenic AQP4 antibodies binding to AQP4 with means of its Fab portion (56). Previous studies have showed that Aquaporumab can decrease brain injury in NMOSD mouse models (57), however, no clinical trials have been carried out to prove such effect of Aquaporumab in humans. In addition, it is reported that another anti-CD20 monoclonal antibody (ublituximab) which contains few fucoses was launched recently. Ublituximab was considered to combine FcγRIIIa more effectively and has better ADCC than rituximab (58). However, during our meta-analysis, we had insufficient clinical data for ublituximab in the treatment of NMOSD. Bevacizumab, a recombinant humanized monoclonal antibody binding to vascular endothelial growth factor (VEGF), can prevent the paracrine and autocrine of VEGF in astrocytes or endothelial cells, which may also be a crucial role in NMOSD treatment (59). The clinical study carried out by Mealy et al. with 10 patients deemed bevacizumab as a safe add-on drug based on high-dose corticosteroids in acute relapse stage (60). Overall, we expected more RCTs about natalizumab, aquaporumab, ublituximab, bevacizumab, or other new monoclonal antibodies about NMOSD treatment to benefit more patients.

To our best knowledge, this is the first meta-analysis carried out for comparing different kinds of monoclonal antibodies, using evidences completely based on RCTs. Previous meta-analyses were focused on rituximab and mainly based on retrospective studies (36, 37, 61, 62). These meta-analyses pooled the data from uncontrolled trials with a heterogeneous dataset which were flawed with the inevitable existence of deviations. Our meta-analysis was different from previous studies. We included all randomized controlled trials which was the best method to divide risk factors into two groups. Although there was another meta-analysis discussing the effectiveness and safety of monoclonal antibodies, however, it only contained 4 RCTs and did not compare monoclonal antibodies with different targets in subgroup analysis (63).

This meta-analysis has few limitations including: (a) Our meta-analysis only pooled seven RCTs (22–27, 29), and therefore collected data was limited. (b) We included 7 RCTs which showed heterogeneity in relapse risk (*I*^2^ = 60%). Further, the sensitivity analysis demonstrated that the consolidated data was stable but the drawback could not be neglected. (c) Present meta-analysis employed different kinds of monoclonal antibodies and some of these included RCTs reserved add-on drugs like immunosuppressors in order to prevent relapse. This might also increase the heterogeneity in our meta-analysis. (d) Compared with the previous meta-analysis about the effectiveness and safety of monoclonal antibodies, we added 3 RCTS but our results were similar to those in the previous one.

## Conclusion

In conclusion, monoclonal antibody therapy could effectively reduce the relapse risk, ARR, EDSS score and serious adverse events in NMOSD patients. During analysis, no significant differences were observed in adverse events and mortality between monoclonal antibody and placebo groups. In subgroup analysis, we detected that eculizumab (anti-complement protein C5 monoclonal antibody) might be the most effective monoclonal antibody for relapse prevention in AQP4-positive patients. In addition, satralizumab and tocilizumab (anti-interleukin-6 receptor monoclonal antibodies) might reduce patients' EDSS score and improve functional recovery more effectively than other types of monoclonal antibodies. Therefore, we conclude that monoclonal antibody therapy for NMOSD is effective and safe, however, more clinical trials are needed to further investigate this issue.

## Data Availability Statement

The raw data supporting the conclusions of this article will be made available by the authors, without undue reservation.

## Author Contributions

ZhW and ZC are the principal investigators. TX and JY designed the study and developed the analysis scheme. SC and YY analyzed the data and performed meta-analyses. TX and JY wrote this article. ZiW and YY revised the manuscript and polished the language. All authors contributed to the article and approved the submitted version.

## Conflict of Interest

The authors declare that the research was conducted in the absence of any commercial or financial relationships that could be construed as a potential conflict of interest.

## Supplementary Material

Supplementary Figure 1 Sensitivity analysis of relapse risk in 7 RCTs.

## Abbreviations

NMOSD: neuromyelitis optica spectrum disorders
RCT: randomized controlled trial
ARR: annualized relapse rate
EDSS: expanded disability status scale
AQP4: aquaporin-4
MOG: myelin oligodendrocyte glycoprotein
AZA: azathioprine
MMF: mycophenolate mofetil
anti-B: anti-B cell monoclonal antibodies
IL-6: anti-interleukin-6 receptor monoclonal antibodies
C5: anti-complement protein C5 monoclonal antibody
VEGF: vascular endothelial growth factor.
