# Supplementary figures and images for: Different Targets of Monoclonal Antibodies in Neuromyelitis Optica Spectrum Disorders: A Meta-Analysis Evidenced From Randomized Controlled Trials
Source: Front Neurol. 2020 Dec 17;11:604445. doi: 10.3389/fneur.2020.604445 (PMC7773932; doi:10.3389/fneur.2020.604445)

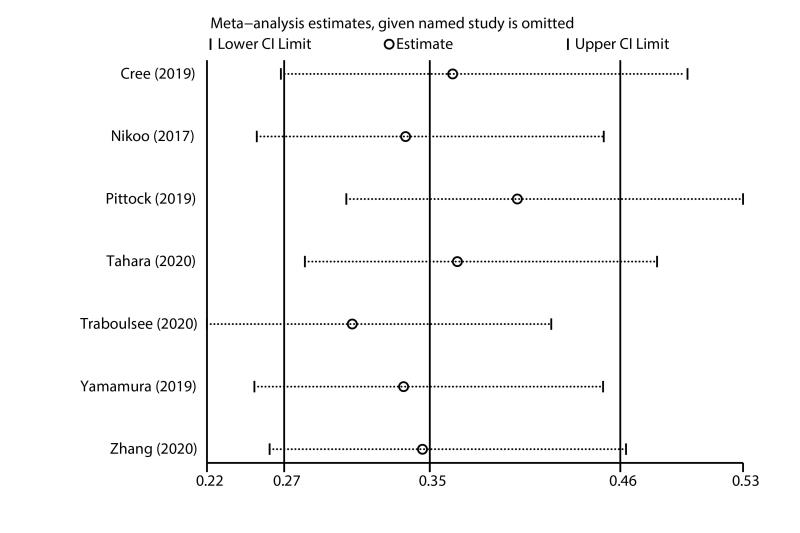

Supplement: Supplementary Figure 1 — Sensitivity analysis of relapse risk in 7 RCTs. [file Image_1.JPEG]
